# Supplementary material for: The Dynamic Risk of COVID-19-Related Events in Vaccinated Healthcare Workers (HCWs) from a Tertiary Hospital in Bucharest, Romania: A Study Based on Active Surveillance Data
Source: Vaccines (Basel). 2024 Feb 11;12(2):182. doi: 10.3390/vaccines12020182 (PMC10891893; doi:10.3390/vaccines12020182)
Supplement: Supplementary file 1 [file vaccines-12-00182-s001.zip › vaccines-2814434-supplementary.pdf]

Summary Data S1. Assessment of proportionality assumption of the risk of COVID-19 events in HCWs in the entire followed-up population

Variables	All COVID-19 events	Symptomatic COVID-19 events	Moderate-to severe COVID-19 events
	p-value	p-value	p-value
Age, Gender			
<29 years	0.056	0.292	0.598
30-39 years	0.109	0.081	0.268
40-49 years	0.367	0.245	0.235
>50 years	0.019	0.093	0.344
Gender	0.671	0.215	0.113
Professional Role			
Physicians	0.326	0.109	0.575
Nurse	0.365	0.997	0.429
Healthcare auxiliary	0.387	0.987	0.801
Other categories	0.622	0.058	0.222
Working in High Risk Department	0.112	0.178	0.098
Vaccination			
Nonvaccinated	0.493	<0.001	0.236
Complete Vaccinated	0.305	0.763	0.975
Vaccinated and Booster	0.248	<0.001	0.144

Summary Data S2. Assessment of proportionality assumption of the risk of COVID-19 events in vaccinated HCWs.

Variables	All COVID-19 events	Symptomatic COVID-19 events	Moderate-to severe COVID-19 events
	p-value	*p*-value	*p*-value
Age, Gender			
<29 years	0.779	0.760	0.889
30-39 years	0.480	0.489	0.975
40-49 years	0.297	0.282	0.927
>50 years	0.489	0.438	0.850
Gender	0.873	0.361	0.026
Professional Role			
Physicians	0.077	0.248	0.888
Nurse	0.228	0.237	0.641
Healthcare auxiliary	0.103	0.473	0.571
Other categories	0.701	0.599	0.998
Working in High Risk Department	0.001	0.002	0.085
Vaccination and hybrid immunity*			
Complete vaccination	0.781	0.791	0.960
Vaccinated and Booster	0.304	0.770	0.654
Hybrid immunity	0.719	0.390	0.841
Hybrid immunity and Booster	0.619	0.458	0.669
